# Endoscopic on-site characterization of a large excavated lesion in longstanding ulcerative colitis using a novel bioinformatic tool

**DOI:** 10.1055/a-2186-5197

**Published:** 2023-11-10

**Authors:** Andrej Wagner, Frieder Berr, Daniel Neureiter, Josef Holzinger, Franz Singhartinger

**Affiliations:** 1 Department of Internal Medicine I, Paracelsus Medical University/Salzburger Landeskliniken, Salzburg, Austria; 2 Department of Internal Medicine I, Paracelsus Medical University/Salzburger Landeskliniken (SALK), Salzburg, Austria; 3 Laboratory for Tumour Biology and Experimental Therapies (TREAT), Institute of Physiology and Pathophysiology, Paracelsus Medical University, Salzburg, Austria; 4 Institute of Pathology, Paracelsus Medical University/Salzburger Landeskliniken (SALK), Salzburg, Austria; 5 Department of Surgery, University Clinics Salzburg, Paracelsus Medical University, Salzburg, Austria; 6 Department of Surgery, Paracelsus Medical University/Salzburger Landeskliniken (SALK), Salzburg, Austria

In a previous study 1 , we reported on a novel tool for real-time visualization and characterization based on bioinformatically enhanced quantitative endoscopic image analysis (BEE) of high definition white-light images. In brief, a grain analysis of selected areas of the endoscopic image is accomplished by thresholding algorithms, and a non-uniformity coefficient is calculated, inspired by soil mechanics and sieve curve terminology 2 .

In longstanding ulcerative colitis, meticulous observation and optical diagnosis is crucial for patient outcomes after surveillance colonoscopy 3 . However, delineating and characterizing suspicious areas and lesions is a major problem due to inflammatory and post-inflammatory changes and interobserver variability 4 . BEE may facilitate optical diagnosis of suspicious lesions and optimize the sensitivity of targeted biopsies, because BEE variables reflect the irregularity and density of vascular and surface structures. Of note, the validated narrow-band imaging magnifying endoscopic classification of colorectal tumors (JNET classification) is based on the optical evaluation of these parameters 5 .

Here we present the case of a 62-year-old woman with longstanding ulcerative colitis and numerous pseudopolyps ( Video 1 ). She was referred to our center for evaluation and endoscopic resection of a large, excavated lesion in the transverse colon. We performed surveillance colonoscopy and characterized the target lesion by BEE. Despite the suspicious morphology of the 3 × 2 cm lesion, we decided not to resect it following visualization of dense and regular vascular and surface patterns obtained by the BEE variables “density” and “non-uniformity coefficient” ( Fig. 1 ) 1 . Accordingly, multiple targeted biopsies showed chronic inflammation and fibrosis, without dysplasia. Therefore, we decided to initiate close endoscopic surveillance rather than resection of the lesion.

Despite a suspicious colonic lesion morphology, we decided not to resect the lesion following visualization of regular vascular and surface patterns, which were obtained by bioinformatically enhanced endoscopy.Video 1

**Fig. 1:**
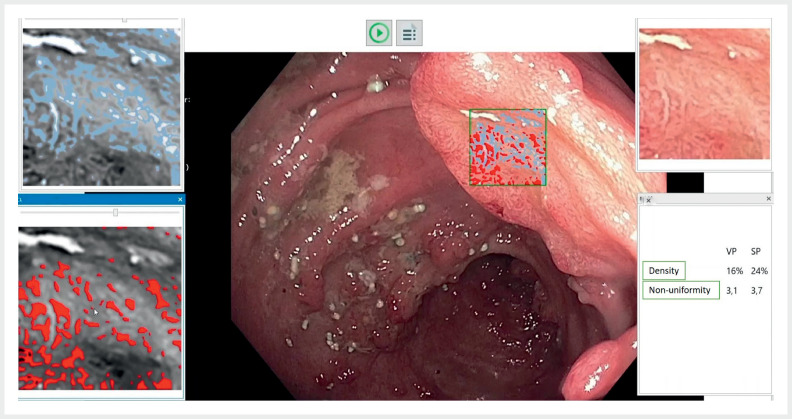
Bioinformatically enhanced endoscopy of the depressed part of the target lesion. After selecting an area of interest, surface structures and microvessels are marked separately (left side). The quantification panel (right lower side) shows dense and regular vascular pattern (VP, density 16%, non-uniformity coefficient <10) and surface pattern (SP, density 24%, non-uniformity coefficient <10).

In this first clinical application, BEE showed promise as a tool for endoscopic characterization of lesions during surveillance endoscopy. We conclude that BEE could support the on-site assessment of colonic lesions in routine endoscopy and underpin treatment decisions. Prospective clinical studies are needed.

Endoscopy_UCTN_Code_CCL_1AD_2AD

## Notes

Endoscopy E-Videos https://eref.thieme.de/e-videos
*E-Videos* is an open access online section of the journal *Endoscopy* , reporting on interesting cases and new techniques in gastroenterological endoscopy. All papers include a high-quality video and are published with a Creative Commons CC-BY license. Endoscopy E-Videos qualify for HINARI discounts and waivers and eligibility is automatically checked during the submission process. We grant 100% waivers to articles whose corresponding authors are based in Group A countries and 50% waivers to those who are based in Group B countries as classified by Research4Life (see: https://www.research4life.org/access/eligibility/ ). This section has its own submission website at https://mc.manuscriptcentral.com/e-videos .
